# Ticks on the turf: investigating the presence of ixodid ticks on and around football fields in Germany

**DOI:** 10.1007/s10493-021-00628-0

**Published:** 2021-06-09

**Authors:** Olaf Kahl, Daniel Kämmer, Ingrid Bulling, Martin Komorek, Christof von Eiff, Claudius Malerczyk

**Affiliations:** 1 tick-radar GmbH, 10555 Berlin, Germany; 2 Pfizer Pharma GmbH, Medical and Scientific Affairs Vaccines Germany, Berlin, Germany

**Keywords:** Ticks, *Ixodes*, *Dermacentor*, Tick exposure, Football, Germany

## Abstract

*Ixodes ricinus* is the most abundant tick species and an important vector of pathogens in Germany and in large parts of Europe. A few other ixodid tick species, e.g., *Dermacentor reticulatus*, may also be of eco-epidemiological relevance. As ticks are not only found in natural but also in suburban areas (parks, gardens), the present study investigated whether ticks occur on and near football grounds thus posing a potential risk to players and visitors. Thirty-two football grounds from all 16 German federal states were selected, mainly situated adjacent to a green area (forest, park). Ticks were collected by the conventional flagging method in spring 2018, and nymphs and adults were counted and morphologically determined. Altogether 807 nymphal and adult ticks were collected from 29 football grounds: 714 *I. ricinus*, 64 *Ixodes inopinatus*, 2 *Ixodes frontalis*, 24 *Ixodes* sp. ticks, and 3 *D. reticulatus*. *Ixodes inopinatus* was found in 13 out of 16 German states. Three ticks were even found on the turf of two football fields. It can be concluded that ticks occur quite frequently and sometimes in high abundance near football grounds situated close or adjacent to a forest or a park.

## Introduction

Ticks (Acari: Ixodida) are parasitic arachnids with a worldwide distribution that feed on the blood of a wide range of vertebrate hosts from reptilia, small mammals and birds to medium-sized and large wildlife and domestic mammals. Many ixodid tick species are known to transmit viral, bacterial or parasitic pathogens to humans and domestic animals during feeding (Jongejan and Uilenberg 2004).

In Germany, the most common exophilic ixodid tick species is *Ixodes ricinus*, which can be found in varying density throughout the country mainly in forests and forest-like habitats with permanent leaf litter (Petney et al. 2012). *Ixodes ricinus* is the primary vector of the causative agents of Lyme borreliosis and tick-borne encephalitis (TBE) and some other agents of medical or veterinary significance (Rizzoli et al. 2014; Dobler et al. 2020; ECDC 2016). Unfed *I. ricinus* move up on vegetation stems while questing in search of hosts, thus passing humans are also likely to get bitten by ticks. While Lyme borreliosis is widespread across Germany with up to approximately 30% of questing nymphal and adult *I. ricinus* carrying *B. burgdorferi* s.l. (ECDC 2016), TBE virus is geographically unevenly distributed (Robert Koch-Institute 2021). TBE endemic areas are mainly found in the southern parts of Germany, in the federal states Bavaria, Baden-Wuerttemberg, Hesse, Thuringia and Saxony, but human TBE cases also occur sporadically in the northern half.

Both *I. ricinus* and *Dermacentor reticulatus*, another common ixodid tick species occurring in large parts of Germany, have also been reported in urban and suburban environments such as parks and gardens (Maetzel et al. 2005; Starostzik 2015; Hohenheim University 2015; Borşan et al. 2020). Sport fields have not been identified as suitable tick habitat and have been considered free of ticks since the turf is usually kept short. This hypothesis, however, needs further scrutiny.

In Germany, more than 7 million people are members of almost 25,000 football clubs organized under the umbrella of the German Football Association (DFB) (German Football Association 2020), representing by far the highest number for any ballgame, and even sport in general. Football training and matches usually take place outdoors almost all the year round. Outdoor football fields in Germany consist mainly of grass, artificial turf, cinder, or tartan fields. Football is the most popular sport in Germany, and it is not uncommon that hundreds of spectators gather around a football field during a match, and sometimes some might even be present during training. Moreover, it is a common occurrence for players and spectators to fetch drifting balls back to the field.

This study aimed to assess the occurrence of ticks on and around football fields. We investigated whether the common paradigm “Ticks do not occur on regularly trimmed sport fields” is valid.

## Materials and methods

**Fig. 1:**
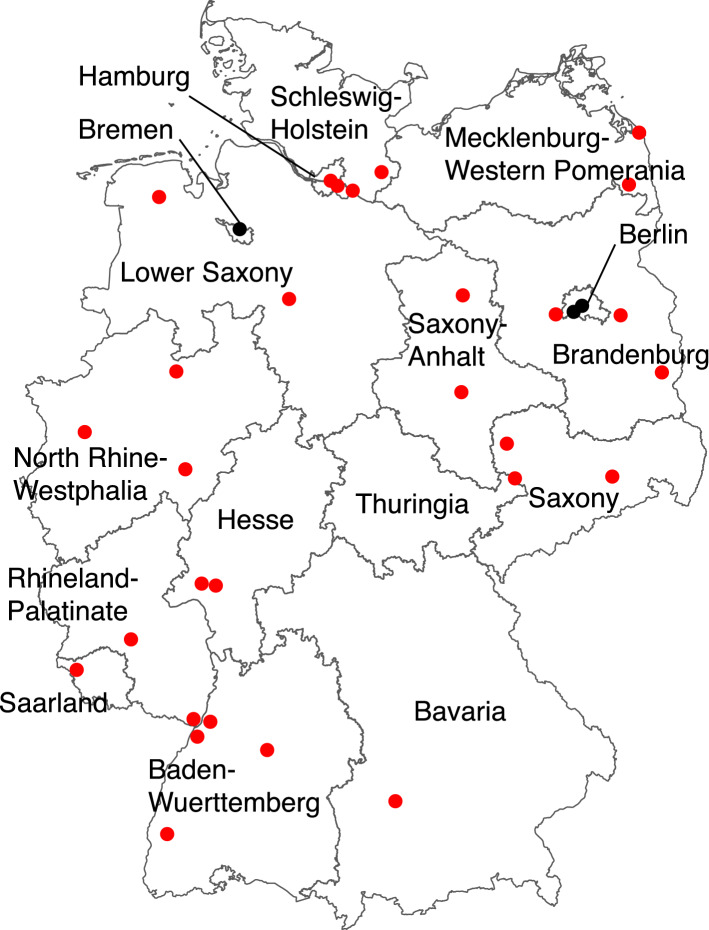
Location of football fields in Germany where efforts were made to collect ticks in April to May 2018. (red circles: with ticks found; black circles: with no ticks found). (Color figure online)

Thirty-two football fields were selected, most of them in the vicinity of green areas or even woodland, one to four from each of the 16 Federal States of Germany, including the City States Berlin, Bremen, and Hamburg (Fig. 1). It would have not been sensible to visit football fields in this study surrounded by streets and houses in a city centre. Flagging was performed in springtime between April 12 and May 18, 2018.

The selected football fields and their close surroundings were screened for questing ticks using conventional dragging with flannel cloth flags. Briefly, a 1.2 × 1.0 m large white flag was drawn twice for a 10-m distance over the lawn of the football field (corresponding to approximately 20 m^2^) and next to the field through ground vegetation or leaf litter (10 drags corresponding to approximately 100 m^2^). Sites were selected up to 40 m away from the field edge by trained professionals. The collected nymphal and adult ticks were counted after each drag, and stored in vials until morphological identification down to stage and species in the laboratory (Nosek and Sixl 1972; Estrada-Peña et al. 2017; Chitimia-Dobler et al. 2018). Occasionally collected *Ixodes* larvae were not considered. A study including *I. ricinus* larvae in Germany should not begin before mid-May because larval activity is usually low in April and early May.

## Results and discussion

After unusually long prevailing winter conditions up to early April the German Weather Service (Deutscher Wetterdienst) reported mean temperatures for April and May 2018 above average in Germany (German Weather Service 2018a, b), hence the ‘tick season 2018’ had a start only in early April, and tick activity was generally rather high during the present investigation.

Altogether, 807 questing ixodid ticks (666 nymphs and 141 adults) were collected in 29 out of 32 football fields and their surroundings (3840 m^2^) assessed (Table 1, Fig. 1). *Ixodes ricinus* was the most common species (n = 714, 88.5% of all ticks), followed by *Ixodes inopinatus* (n = 64 ticks, 8.0%), and *Ixodes frontalis* (two individuals, 0.2%). Twenty-four further ticks (3.0%) belonged to the genus *Ixodes* but could not be determined down to species with certainty because of ambiguous morphology. Also, three *D. reticulatus* adults (0.4%) were retrieved from the vicinity of two football fields in Saxony-Anhalt and Brandenburg (Table 1, Fig. 1).

**Table 1:** Ticks collected on or from the surroundings of football fields in different federal states in Germany in spring 2018

Site no.	Federal State	*Ixodes ricinus*		*Ixodes inopinatus*		*Ixodes frontalis*		*Ixodes* sp.		*D. reticulatus*	Total
		N	A	N	A	N	A	N	A	A	
1	Baden-Wuerttemberg	13	4		2			1	1		21
2		24	13	2	1				1		41
3		2									2
4			1								1
5	Bavaria	1	1								2
6	Berlin										0
7											0
8	Brandenburg	2			2					1	5
9		5	1	1							7
10		48	5	4	2			2	1		62
11	Bremen										0
12	Hamburg	34	3	2				1	1		41
13		6									6
14	Hesse	34	3	2							39
15		4		1							5
16	Lower Saxony	6	10		1						17
17		14	1	3					1		19
18	MWP	17	4		2			1	1		25
19		68	1	2	1			2			74
20	North Rhine-Westphalia	13	18		3		1		2		37
21			1								1
22		133	5	8	1			1	1		149
23	Rhineland-Palatinate	3			1						4
24		16	3								19
25	Saarland	13	2								15
26	Saxony-Anhalt	7		1					2	2	12
27		17	3	6	1				1		28
28	Saxony	32	6	4	4	1					47
29		8	4	1							13
30	Schleswig–Holstein	32	6	1	1			2			42
31		43	2								45
32	Thuringia	18	4	3	1			1	1		28
total		613	101	41	23	1	1	11	13	3	807

*A*, adults; *N*, nymphs; *Loc*, location; *MWP*, Mecklenburg-Western Pomerania

Although the large majority of ticks was found in the vegetation surrounding of football fields, three ticks were found on the turf (on two separate football fields), which was unexpected, given the fact that the turf had been kept short, the leaf litter had been swept away, and the grass was often exposed to direct sunshine. *Ixodes ricinus* is mainly an inhabitant of forest, edges of forest (e.g., pastures in close vicinity of a forest), and forest-like habitat in continental Europe (L’Hostis et al. 1995; Gray et al. 1998; Mémeteau et al. 1998; Kahl et al. unpublished). It is insofar not surprising that this tick species does occur around football fields which verge on a forest or forest-like habitat where *I. ricinus* may find suitable hosts and might be able to pass all the necessary off-host developmental steps in the vegetation litter (e.g., embryonic development, moulting).

The results of this study confirm previous findings that report *I. ricinus* as the most common tick species in Germany (Petney et al. 2012). *Ixodes inopinatus* was present in 21 out of 32 study sites in 13 out of 16 German federal states and seems to be a rather common occurrence in Germany, thus confirming the results of Chitimia-Dobler et al. (2018) and Hauck et al. (2019). However, the distribution and abundance of *I. inopinatus* in Germany need to be further investigated including genetical determination, which was out of scope of the present study. In addition, the eco-epidemiological significance of *I. inopinatus* as a possible vector of, e.g., *B. burgdorferi* s.l. or TBE virus, is currently unknown and needs further research.

The bird tick, *Ixodes frontalis*, seems to be more common in Germany than previously thought (Drehmann et al. 2019; Kahl et al. 2019), thus the findings of single questing individuals of this tick species are not unusual. Nevertheless, such findings need careful morphological determination, which may not be the case in every study. Otherwise especially the immature stages can be easily overlooked and erroneously taken as *I. ricinus*.

Also, the finding of single questing adult *D. reticulatus* is not unexpected because, in contrast to *I. ricinus*, this species prefers more open landscapes rather than dense forest (Rubel et al. 2020). This tick species rarely bites humans in Germany, still its occurrence is especially of veterinary relevance since *D. reticulatus* is a proven vector of *Babesia canis*, which can cause serious disease in dogs (Gray et al. 2019).

It can be concluded that ticks occur quite frequently, and sometimes in high abundance around and occasionally even on football fields situated close or adjacent to a forest or a forest-like area in Germany. Since the football fields and the surrounding environments (parks, forests) are used by many people and their pets for recreational purposes, the outcome of this study could help raise awareness among football players, staff, and spectators concerning the risk of tick encounters in such areas and thus the need for precautionary measures (e.g. use of repellents, careful check of body and clothes timely after exposure). This combination of measures is, for example, highly efficient against an infection with *B. burgdorferi* sensu lato genospecies, which can cause Lyme borreliosis and occur all over Germany in *I. ricinus*. In areas where TBE virus is present (Robert Koch-Institute 2021; Walter et al. 2020) such measures should include vaccination against TBE, the most reliable measure of individual TBE prevention.
